# Postural sway is not affected by estrogen fluctuations during the menstrual cycle

**DOI:** 10.14814/phy2.15693

**Published:** 2023-05-22

**Authors:** Sasha Reschechtko, Thuy Ngoc Nguyen, Michelle Tsang, Kristine Giltvedt, Mark Kern, Shirin Hooshmand

**Affiliations:** ^1^ School of Exercise and Nutritional Sciences San Diego State University San Diego California USA

**Keywords:** balance, estrogen, menstrual cycle

## Abstract

When people stand still, they exhibit a phenomenon called postural sway, or spontaneous movement of the body's center of pressure, which is related to balance control. In general females show less sway than males, but this difference only begins to appear around puberty, pointing to different levels of sex hormones as one potential mechanism for sway sex differences. In this study, we followed cohorts of young females using oral contraceptives (*n* = 32) and not using oral contraceptives (*n* = 19), to investigate associations between estrogen availability and postural sway. All participants visited the lab four times over the putative 28‐day menstrual cycle. At each visit, we performed blood draws to measure plasma estrogen (estradiol) levels, and tests of postural sway using a force plate. During late follicular and mid‐luteal phase, estradiol levels were lower in participants using oral contraceptives (mean differences [95% CI], respectively: −231.33; [−800.44, 337.87]; −613.26; [−1333.60, 107.07] pmol/L; main effect *p* < 0.001), reflecting expected consequences of oral contraceptive use. Despite these differences, postural sway was not significantly different between participants who were using oral contraceptives and participants who were not (mean difference: 2.09 cm; 95% CI = [−1.05, 5.22]; *p* = 0.132). Overall, we found no significant effects of the estimated menstrual cycle phase—or absolute levels of estradiol—on postural sway.

## Graphical abstract

Estrogen may change the mechanical properties of connective tissues, which could affect balance. We followed two cohorts of female participants—one naturally cycling and one on oral contraceptive—over four visits during the menstrual cycle to test postural sway and plasma estradiol level. We found no difference in balance over the course of the menstrual cycle in either cohort or between cohorts, despite phasic differences in estrogen levels between cohorts.

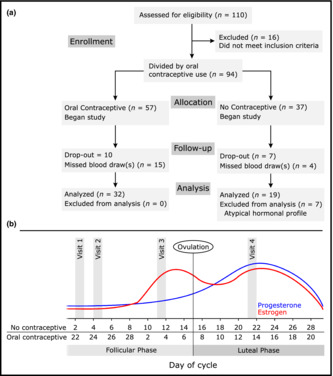

## 1 INTRODUCTION

Estrogen plays a variety of roles in extragonadal tissues. In addition to its wide‐ranging effects on bone (Cutler, 1997; Kalervo Väänänen & Härkönen, 1996; Riggs, 2000), estrogen plays a role in the formation, maintenance, and function of muscle, connective tissue, and neural tissue (Chidi‐Ogbolu & Baar, 2019; Cui et al., 2013; Hansen, 2018; Leblanc et al., 2017). During the menstrual cycle estrogen levels can vary by orders of magnitude (Buchanan et al., 1986; Denver et al., 2019). Despite these large fluctuations in estrogen levels and estrogen's widespread effects, it remains unclear whether estrogen availability affects motor behaviors or performance.

One important motor ability is balance or our ability to maintain an upright posture without falling over. Postural sway—a measure of spontaneous shifts of a person's center of mass as they stand stationary—is consistently lower in females compared to males, but this difference does not appear until around puberty and is not accounted for by other likely covariates like height or weight (Goble & Baweja, 2018a, 2018b; Moran et al., 2020). While postural sway is not the same as balance and may reflect multiple neural processes (Carpenter et al., 2010; Latash & Zatsiorsky, 2016; Mochizuki et al., 2006), it is interpreted as related to balance in that people who show more postural sway are considered to have worse balance and be more likely to fall (Fernie et al., 1982; Johansson et al., 2017).

Balance depends on both sensory and motor factors (Mancini & Horak, 2010; Shumway‐Cook & Horak, 1986). Estrogen can alter the structural properties of connective tissues by changing the amount of collagen incorporated into them, the formation of collagen‐elastin crossbridge structures, and connective tissue metabolism (Chidi‐Ogbolu & Baar, 2019; Hansen, 2018; Leblanc et al., 2017). These changes could affect the mechanical connections between sensory receptors and muscles and could be reason for some reports of changes in reflex properties during the menstrual cycle (Casey et al., 2014). However, observations of functional changes due to altered mechanical properties in vivo during the menstrual cycle do not show a consensus. While some studies have indicated changes in some connective tissue and muscle properties over the menstrual cycle (Lee et al., 2013; Maruyama et al., 2022; Petrofsky & Lee, 2015; Shagawa et al., 2021; Yim et al., 2018), others have not reported functional changes on that timescale (Bryant et al., 2008; Burgess et al., 2009; Ericksen & Gribble, 2012; Kubo et al., 2009).

A number of studies have reported that balance changes over the course of the menstrual cycle (Darlington et al., 2001; Maged et al., 2017; Mokošáková et al., 2018; Petrofsky & Lee, 2015; Sung & Kim, 2018). In conjunction with observations about the timing and prevalence of certain knee and ankle injuries (Wojtys et al., 2002), these findings have been interpreted as indicative of increased injury risk and been taken as evidence supporting the prophylactic use of oral contraceptives to decrease musculoskeletal injury risk in female athletes. However, a number of the cited studies use small participant cohorts (≤15) (Darlington et al., 2001; Fridén et al., 2003; Mokošáková et al., 2018; Petrofsky & Lee, 2015) and some use balance measures that are difficult to replicate or interpret because they are scores related to proprietary testing apparatus (Darlington et al., 2001; Fridén et al., 2003; Maged et al., 2017; Yim et al., 2018) instead of physical measures of sway like those we use in the present study (e.g., distance the center of pressure moves). Finally, these studies rarely measure serum hormone levels to investigate hormone level as a mechanism. Here, we analyze both postural sway and hormone levels four times over the course of the menstrual cycle in 19 young female participants who reported natural menstrual cycling as well as 32 young females taking oral contraceptives.

## 2 METHODS

### 2.1 Participants

We recruited healthy young female participants who were either using oral contraceptives or not using oral contraceptives to participate in this study. Participants were required to be between 18 and 25 years old, have a BMI between 18 and 32 kg/m^2^, be non‐smoking, consume fewer than two alcoholic drinks per day, and not be pregnant or lactating. We also required that participants using oral contraceptives had used them for at least 1 year and no more than 5 years and those not using oral contraceptives had not used them for at least 3 months prior to their enrollment. Potential participants were given a questionnaire regarding their menstrual history and oral contraceptive use; based on their answers, participants were divided into a cohort using oral contraceptives and a cohort who were not using oral contraceptives (naturally cycling). These participants were part of a larger study investigating the effects of oral contraceptives on bone health. All participants provided written informed consent. We conducted this study in accordance with procedures approved by the San Diego State Institutional Review Board's Human Research Protection Program.

### 2.2 Testing protocol and quantifying menstrual cycle

Following an initial lab visit for familiarization and screening, we scheduled each participant to make four visits for blood collection and balance testing. For participants who were not using oral contraceptives, these visits corresponded to menstrual cycle days 2, 4, 11, and 21 (first day of follicular phase, early follicular phase, ovulation, and mid‐luteal phase, respectively). For participants using oral contraceptives, these visits corresponded to pill pack days 21 (start of placebo pills), 24, 3, and 13. We chose these intervals for participants on oral contraceptives to coincide with the start of the estrogen‐free pill phase of the oral contraceptives (Day 21) and to match the time between visits in the naturally cycling cohort. Participants always visited the laboratory in the same order: their first testing visit corresponded to cycle day 2 (21 for oral contraceptive users)—participants contacted the study group to schedule their first test as soon as menses began—and the last visit was Day 21 (13 for oral contraceptive users). We asked participants to fast for 10 h before their scheduled visits and we instructed oral contraceptive users who consumed their pill in the morning to wait until after the blood draws. We centrifuged blood samples immediately after blood draws for 15 min at 4000 × ***g*** at 4°C and subsequently stored the samples at −80°C until analysis.

### 2.3 Evaluating balance

We evaluated participants' balance using the BtrackS Balance Test (BBT; Balance Track Systems, Inc.). The BtrackS system incorporates a standardized force plate and data collection protocol to quantify postural sway during quiet standing. Postural sway results from the natural, spontaneous motion of a person's center of mass as they stand stationary and reflects the combination of neural outputs and sensory inputs that govern balance. Balance is interpreted as inversely related to postural sway: a person who displays more postural sway (meaning their center of mass moves more) is considered to have worse balance.

The BBT testing protocol involves recording postural sway during three, 20 s trials following one familiarization trial. During these trials, participants are instructed to stand as still as possible on the BtrackS force plate with their eyes closed and feet shoulder width apart. The BtrackS force plate records the center of pressure (the projection of the center of mass onto the ground) at a rate of 25 Hz. Results from the BBT have been validated against laboratory‐quality force plates (Goble et al., 2018; O'Connor et al., 2016; Richmond et al., 2018). The outcome measure of the BBT (“BBT Score”) is the average length, in centimeters, of the path that the center of pressure covers during the three trials. While the precise link between balance and postural sway is unknown (Latash & Zatsiorsky, 2016), extensive normative data are available for BBT scores and this balance testing paradigm is unaffected by practice or prior experience which could arise from testing participants multiple times over the course of the study (Hearn et al., 2018).

### 2.4 Measuring sex hormones and hormonal inclusion criteria

We measured estradiol, which is the most biologically active and most prevalent form of estrogen, as well as progesterone. We analyzed the level of estradiol in plasma in each sample using ultra‐sensitive estradiol ELISA kits (ALPCO). These ultra‐sensitive ELISA kits have a sensitivity of 5.14 pmol/L with a range of 5.14–734.21 pmol/L. For samples that exceeded the upper limit of the ultra‐sensitive ELISA kits, we then used standard ELISA kits (ALPCO) which have a sensitivity of 36.71 pmol/L with a range of up to 11,747 pmol/L. To further analyze participants' hormone profiles, we also measured serum progesterone levels. The ELISA kits (ALPCO) we used to measure plasma progesterone levels have a sensitivity of 0.32 nmol/L and a range of 0.95–191 nmol/L.

We imposed additional inclusion criteria on participants who were not using oral contraceptives to ensure they showed a typical hormonal profile during the menstrual cycle. We only included participants who (1) showed peak progesterone levels at Visit 4 (mid‐luteal phase), and (2) showed an increase in estradiol levels from Visit 1 (early follicular phase) to Visit 3 (late follicular phase). We imposed these criteria to ensure a participants showed a relatively regular menstrual cycle; it has been suggested that a major source of variability in previous literature investigating the menstrual cycle is the inclusion of females with atypical menstrual cycles in supposedly eumenorrheic groups (Dam et al., 2022). Although excluded participants' data are not included in the current analyses, all data are available in a linked repository.

### 2.5 Statistical analysis

To test the effect of the menstrual cycle and oral contraceptive use on estradiol levels, we used linear mixed‐effects models. We log‐transformed estradiol level for normality, as assessed visually via QQ‐plots. We used the same methods to assess the effect of the menstrual cycle and oral contraceptive use on the BBT score to investigate postural sway. We report Bayes Factors (BF_10_) for additional interpretive value; unlike large *p*‐values, small Bayes Factors can be interpreted as evidence for the null hypothesis (Kass & Raftery, 1995), and large Bayes Factors can also indicate that a study is properly powered. These tests use day of menstrual cycle as a proxy for estradiol level, so we also directly investigated whether there was an association between estradiol level and balance by running repeated‐measures linear correlation on estradiol level and BBT score.

We used JASP (version 0.16.3, JASP Team, 2022) to perform our statistical analyses. We analyzed the effect of the day of the menstrual cycle on hormone levels and postural sway using linear mixed‐model analysis. We constructed the linear mixed‐effects models using the Linear Mixed Models workflow in JASP with Visit number (4 levels) and Contraceptive status (2 levels) treated as fixed effects and individual participants as random factors for which we estimated random intercepts but not slopes. We performed the same analyses in a Bayesian framework with the Bayesian ANOVA workflow in JASP treating individual participants as random factors and default assumptions. We performed repeated‐measures correlations using the *rm_corr* command in the pingouin package (Vallat, 2018) for Python.

## 3 RESULTS

**FIGURE 1:**
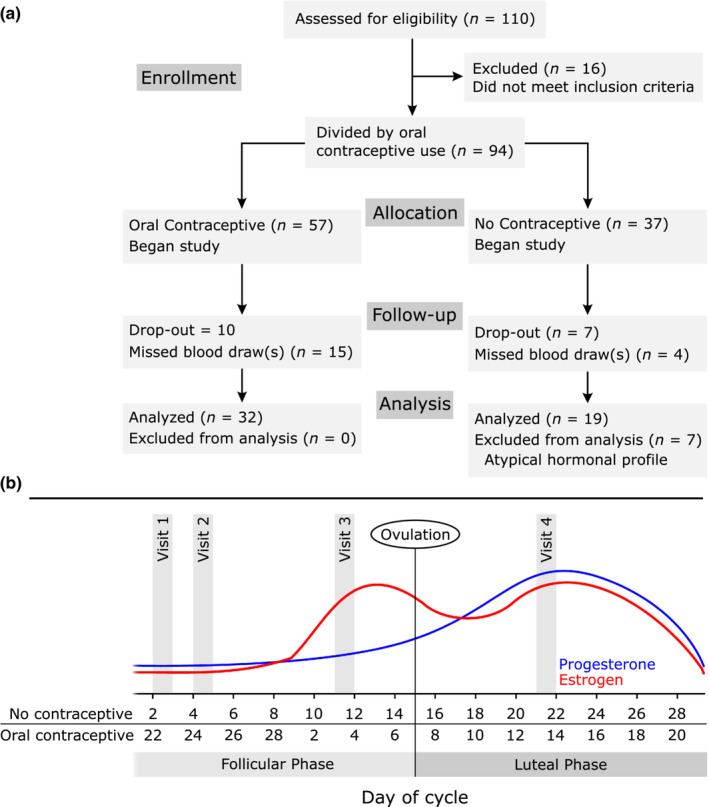
Experimental design and participant inclusion. (a) Workflow indicating participants considered for analysis and exclusion criteria. (b) Schedule of visits and “typical” menstrual cycle hormonal levels adapted from (Reed & Carr, 2000). Testing intervals were the same for participants using oral contraceptives and those not using oral contraceptives but for those using oral contraceptives testing was chosen to start on the first day pill pack placebo.

Fifty‐eight of the participants completed full sets of both balance tests and blood draws (32 using oral contraceptives and 26 not using oral contraceptives). This mainly occurred because some participants had not yet fully healed from their first blood draw when they returned for their second blood draw (2 days later) and were therefore unable to undergo that blood draw. After imposing additional constraints on hormonal profile in participants who were not using oral contraceptives (described in Section 2), we included 19 of 26 participants not using oral contraceptives in our analyses. Physical characteristics and demographics of the participants we included in analysis are provided in Table 1 and a visualization of testing schedule and participant recruitment, retention, and analysis is shown in Figure 1. After imposing inclusion criteria based on sex hormone levels, 19/26 of our participants not using oral contraceptives were eligible for inclusion in analysis.

**TABLE 1:** Demographic information for participants included in the study.

	No contraceptive (*n* = 19)	Oral contraceptive (*n* = 32)
Age (years)		
Mean	21.89	21.34
SD	2.08	1.73
Height (cm)		
Mean	162.18	163.78
SD	6.02	5.82
Weight (kg)		
Mean	62.27	61.02
SD	5.82	7.65
Demographics		
White	10	20
Hispanic	7	4
Asian	2	4
Black	0	2
Other	0	2

### 3.1 Confirmation of decreased estradiol levels in oral contraceptive users

Across all visits and participants, estradiol levels in participants using oral contraceptives ranged from 0.50 to 4837.35 pmol/L; in participants not using oral contraceptives, estradiol levels ranged from 19.64 to 5685.12 pmol/L. Visit‐by‐visit mean values and SD are shown in Table 2. These levels and averages are similar to those reported in previous studies (Bryant et al., 2008; Buchanan et al., 1986; Denver et al., 2019).

**TABLE 2:** Summary statistics for estradiol level and postural sway at each visit in both cohorts of participants. Mean differences computed for Contraceptive group—no contraceptive group

	Contraceptive	No contraceptive	Mean difference
	Mean (SD)	Mean (SD)	[95% CI]
Estradiol (pmol/L)			
Visit 1	366.92 (766.55)	412.99 (723.07)	−46.07 [−472.15, 380.00]
Visit 2	464.55 (936.61)	477.77 (950.92)	−13.21 [−547.87, 521.45]
Visit 3	493.90 (883.54)	725.23 (1207.54)	−231.33 [−800.44, 337.78]
Visit 4	532.61 (1052.76)	1145.88 (1641.29)	−613.26 [−1333.60, 107.07]
Postural sway (cm)			
Visit 1	23.79 (6.88)	21.31 (4.16)	2.47 [−0.87, 5.81]
Visit 2	23.65 (5.98)	20.84 (4.42)	2.81 [−0.26, 5.88]
Visit 3	23.20 (5.64)	22.31 (4.77)	0.88 [−2.14, 3.90]
Visit 4	23.60 (6.06)	21.42 (5.01)	2.18 [−1.04, 5.40]

*Note*: Statistics were run on log‐transformed estradiol levels, but actual values are reported here for interpretability.

**FIGURE 2:**
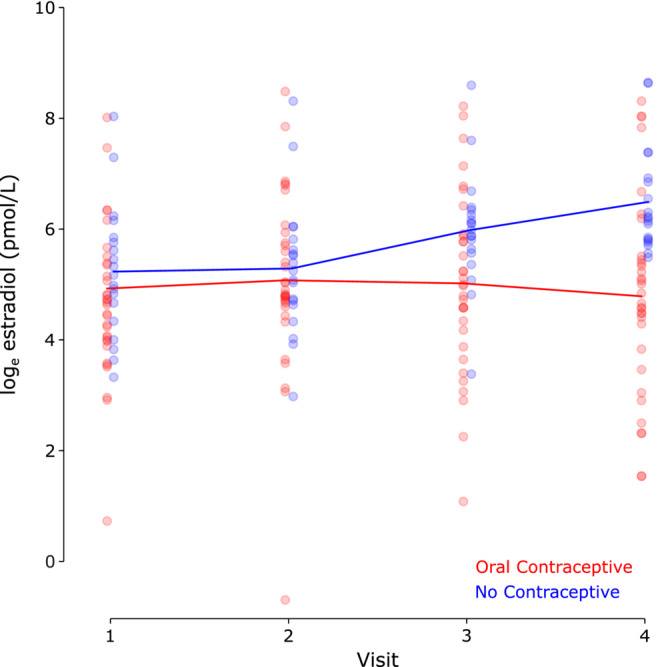
Estradiol levels. Plasma estradiol levels as measured via blood draw during the four experimental visits. Blue: females not using oral hormonal contraceptives (*n* = 19); red: females using oral hormonal contraceptives (*n* = 32). Each dot represents a single participant. Estradiol data are log‐transformed for analysis and visualization. In the participant cohort using oral contraceptives, visit days correspond to days 2, 4, 11, and 21 of the putative 28‐day menstrual cycle.

First, we confirmed that oral contraceptives altered estradiol. We found a significant interaction between day of menstrual cycle and oral contraceptive use (*F*_3,174_ = 14.68; *p* = 2.05e^−8^; BF_10_ = 58.92), indicating that estradiol levels were higher during Days 11 and 21 than Days 2 and 4 for participants not using oral contraceptives. In contrast, estradiol levels did not vary significantly over the course of the menstrual cycle for participants using oral contraceptives, so estradiol levels in the cohort using oral contraceptives were lower than in the cohort not using oral contraceptives during Days 11 and 21. These results are shown in Figure 2.

### 3.2 Postural sway is unaffected by menstrual cycle

**FIGURE 3:**
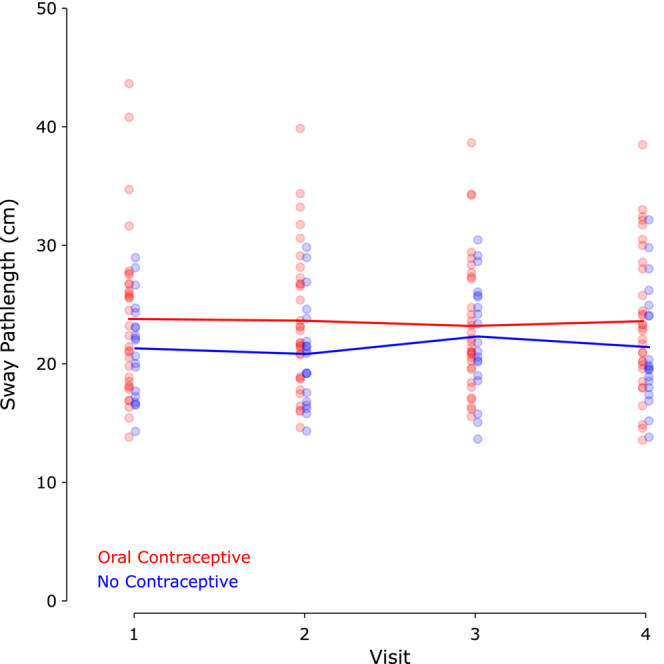
Postural sway. Postural sway, quantified as the average pathlength of the center of pressure during three 20 s trials of eyes‐closed quiet standing. Blue: females not using oral contraceptives (*n* = 19); red: females using oral contraceptives (*n* = 32).

We next investigated whether postural sway varied over the course of the menstrual cycle for the two cohorts of female participants. We again used a linear mixed‐effects model to investigate the effect of day of the menstrual cycle and contraceptive use on postural sway. This analysis did not show any significant effect of day of the menstrual cycle (*F*_3,147_ = 0.16; *p* = 0.92; BF_10_ = 0.026) or of contraceptive use (*F*_1,49_ = 2.35; *p* = 0.13; BF_10_ = 0.85), additionally, there was no significant interaction between contraceptive use and day of menstrual cycle. Across days, the mean difference between participants who used oral contraceptives and those who did not was 2.09 cm (95% CI = [−1.05, 5.22]); day‐by‐day means and standard deviations (as well as mean differences and 95% CI) for postural sway are shown in Table 2. The minimum meaningful difference for this test is 5 cm (Goble et al., 2016). Compared to previously published normative data using this postural sway paradigm, all of these values are between the 30th and 50th percentiles for women ages 15–29 (Goble & Baweja, 2018b). These results are shown in Figure 3.

### 3.3 Estradiol level is minimally associated with postural sway

**FIGURE 4:**
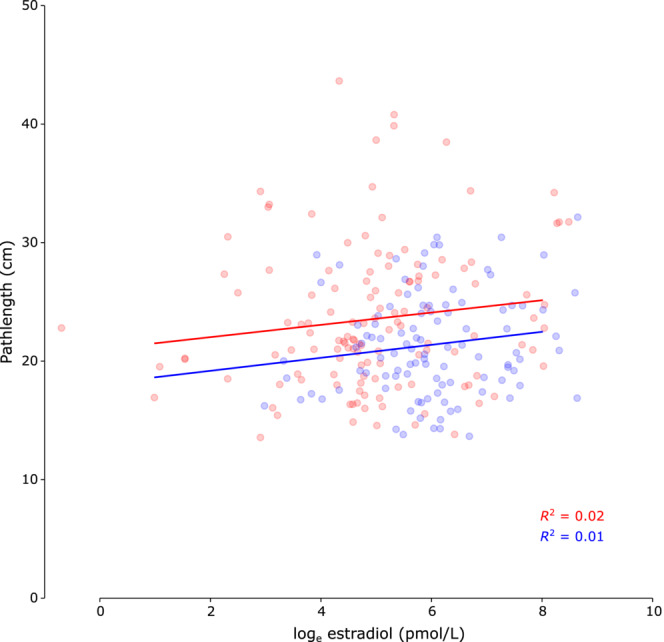
Comparison of estradiol and postural sway. Scatterplot estradiol level (log‐transformed) and postural sway (pathlength of center of pressure excursion) for females using oral contraceptives (red; *n* = 19) and females not using oral contraceptives (blue; *n* = 32). Repeated‐measures correlations were not significant in either cohort. Solid lines represent best linear fits and *R* ^2^ values for these fits are provided.

Finally, we directly tested whether estrogen level was associated with postural sway using repeated‐measures correlation (Bakdash & Marusich, 2017; Vallat, 2018) of estradiol level with postural sway. The repeated‐measures correlation between estradiol level and postural sway was not significant for participants using oral contraceptives (*r* = 0.08; *p* = 0.46; 95% CI = [−0.12, 0.27]) or for participants not using oral contraceptives (*r* = 0.10; *p* = 0.44; 95% CI = [−0.16, 0.35]). These data are shown in Figure 4. Additionally, we compared the two cohorts in a mixed linear effects model incorporating the actual estradiol levels rather than visit days (visit days were used in the previously described analyses). Model slope (indicating the estimated effect of estradiol level on postural sway) in participants not using oral contraceptives was 0.40 (95% CI = [−0.62, 1.41]) and 0.49 (95% CI = [−0.27, 1.25]) in participants using oral contraceptives.

## 4 DISCUSSION

Our measures of balance do not provide evidence that postural sway changes over the course of the menstrual cycle (and BF_10_ < 0.1 is often interpreted as moderate evidence for the null hypotheses, for example, no differences due to menstrual cycle fluctuations). We found no significant differences in postural sway between participants using oral contraceptives (who had lower levels of estradiol) and participants who were not using oral contraceptives, but a larger sample size might be required to elucidate this point further (BF_10_ ≈ 1). When we compared estradiol levels to participant sway across all participants, we found no evidence that varying levels of estradiol affect postural sway in people using oral contraceptives.

### 4.1 Previous association of balance and hormone levels

Previous studies report conflicting findings about whether balance‐related measures change over the course of the menstrual cycle. Some groups have reported changes in balance over the course of the menstrual cycle during “more challenging” tests, for example, eyes closed in combination with unstable surfaces and tandem foot position in which one foot is in front of the other (Petrofsky & Lee, 2015). While it is possible we would have seen differences if we had used other balance‐related testing protocols, there are also studies that report changes in balance metrics using methods that are similar to those we used (eyes closed and standing on a firm surface) over the course of the menstrual cycle (Mokošáková et al., 2018) as well as studies that do not report changes using dynamic balance tasks (Ericksen & Gribble, 2012).

While we might have observed significant differences over the course of the menstrual cycle if we had used different balance measures, but we elected to use a well‐documented balance test with a large amount of normative data instead. Most previous studies investigating menstrual cycle‐related fluctuations in balance control with objective measurement of balance have used custom instrumentation and reported measures in arbitrary units (Darlington et al., 2001; Fridén et al., 2003) or units that are specific to the balance testing apparatus (Maged et al., 2017; Sung & Kim, 2018). This makes it difficult to link experimental findings to any physical quantity to understand behavior. As such, we think that the easily replicable testing paradigm and interpretability of postural sway as a straightforward measure of postural control in the present study are important contributions.

### 4.2 Different timescales of fluctuations in estrogen levels and motor adaptation

Hormone levels vary cyclically over the course of the menstrual cycle, and they also vary over lifetime via aging and with long‐term exposure to exogenous hormones, for example via oral contraceptives. This study tested both those short‐term fluctuations in the cohort of participants not using oral contraceptives and longer‐term fluctuations between the two participant cohorts. Although we did not find changes in postural control in either case, there is stronger evidence against changes due to short‐term fluctuations according to the day of the menstrual cycle than during long‐term fluctuations between females on and off oral contraceptives.

In the context of connective tissue, several studies have not found changes in the structural properties of connective tissue over the course of the menstrual cycle (Burgess et al., 2009, 2010; Kubo et al., 2009). However, comparisons of males and females show differences in measures related to connective tissue properties (Beynnon et al., 2005; Shafiei et al., 2016), and some similar results are reported when comparing apparently eumenorrheic females and those taking oral contraceptives (Bryant et al., 2008). These latter findings agree with changes that would be expected due to changes in connective tissue metabolism (Chidi‐Ogbolu & Baar, 2019; Hansen, 2018; Leblanc et al., 2017) although much of the work in this area has not been performed in humans. A few studies in humans have shown that, even though the physical properties of tissues change, other types of voluntary behavior like maximum voluntary force production does not change over the course of the menstrual cycle either (Bryant et al., 2008; Elliott et al., 2005). The central nervous system is able to compensate for numerous changes in the body over the course of development and aging to support our ability to successfully interact with the world over lifespan. While it may seem surprising that the central nervous system could adapt to changes in the body so rapidly, motor adaptation paradigms routinely report adaptation on the timescale of hours (Maeda et al., 2020; Weiler et al., 2019) even in the context of supposedly “hard wired” responses like short‐latency reflexes. Because we observed changes in plasma estradiol (which may have led to changes in mechanical properties of tissues) but no associated changes in balance, our results suggest that postural control could similarly quickly adapt to such systemic changes over the course of days during the menstrual cycle.

### 4.3 Limitations

Menstrual cycle atypicality is common among females who are not using hormonal contraceptives. Our original study cohort included participants who may have had luteal phase‐deficient or anovulatory cycles, and we subsequently excluded seven participants who were not using oral contraceptives. While exclusion of these participants did not qualitatively change any statistical outcomes, other factors including the assumption of a putative 28‐day menstrual cycle for scheduling participant visits could still have affected our measures, especially as they relate to changes in sway during the menstrual cycle (as opposed to those linked to estradiol level). Additionally, we did not record participants' levels of physical activity, which could also affect menstrual or balance outcomes.

Postural control is difficult to define, and postural sway is not a direct measure of balance. It is possible that our measure of sway did not capture some aspect of balance that is associated with changes in hormone levels. Additionally, in the context of sports injuries, the series of events that lead to an injury (like a rapid change in direction) may not be closely linked with the factors measured via postural sway. Because of this, it is possible that there are balance‐ or injury‐related measures that would be sensitive to changes in hormone level but which we did not capture using postural sway.

### 4.4 Concluding comments

While previous studies have investigated the effects of the menstrual cycle on balance, our study makes the following important contributions. First, we quantified balance using an easily understood measure (postural sway) which is reported in common physical units and can be compared to normative data from more than 16,000 people (Goble & Baweja, 2018b). Second, we recruited a large cohort of female participants whom we followed to obtain longitudinal data at four specific timepoints related to the menstrual cycle. Finally, we measured both plasma estradiol levels and postural sway in the same participants during the same visits, allowing us to directly investigate the potential relationship between hormone levels and balance, rather than using reported menstrual cycle phase as a proxy for hormone levels which vary widely among individuals.

## AUTHOR CONTRIBUTIONS

Sasha Reschechtko drafted the manuscript. Sasha Reschechtko, Mark Kern, and Shirin Hooshmand edited and revised the manuscript. Mark Kern and Shirin Hooshmand designed the research. Thuy Ngoc Nguyen, Kristine Giltvedt, Mark Kern, and Shirin Hooshmand collected the data. Sasha Reschechtko and Thuy Ngoc Nguyen analyzed the data. Sasha Reschechtko and Thuy Ngoc Nguyen interpreted the findings.

## FUNDING INFORMATION

Data collection for this study was supported by the California Prune Board Grant G00012885 to M.K. and S.H.

## CONFLICT OF INTEREST STATEMENT

The authors declare no conflicts of interest, financial or otherwise.

## ETHICS STATEMENT

This study including all experimental procedures was approved by the San Diego State Institutional Review Board’s Human Research Protection Program.

## Data Availability

The data that support the findings of this study, as well as additional data not included in the analyses presented here, are openly available in Open Science Framework at https://osf.io/vubds/ DOI: 10.17605/OSF.IO/VUBDS
